# Effects of heteroatom substitution in spiro-bifluorene hole transport materials†
†Electronic supplementary information (ESI) available. See DOI: 10.1039/c6sc00973e


**DOI:** 10.1039/c6sc00973e

**Published:** 2016-05-03

**Authors:** Zhao Hu, Weifei Fu, Lijia Yan, Jingsheng Miao, Hongtao Yu, Yaowu He, Osamu Goto, Hong Meng, Hongzheng Chen, Wei Huang

**Affiliations:** a School of Advanced Materials , Peking University Shenzhen Graduate School , Shenzhen 518055 , China . Email: menghong@pkusz.edu.cn; b State Key Laboratory of Silicon Materials , MOE Key Laboratory of Macromolecular Synthesis and Functionalization , Department of Polymer Science & Engineering , Zhejiang University , Hangzhou 310027 , China; c Key Lab for Flexible Electronics & Institute of Advanced Materials , Jiangsu National Synergistic Innovation Center for Advanced Materials (SICAM) , Nanjing Tech University , 30 South Puzhu Road , Nan-Jing , P. R. China

## Abstract

Three new spirofluorene-based hole transport materials, Spiro-S, Spiro-N, and Spiro-E, are synthesized by replacing the *para*-methoxy substituent in 2,2′,7,7′-tetrakis(*N*,*N*-di-*p*-methoxyphenylamine)-9,9′-spirobifluorene (Spiro-MeOTAD) with methylsulfanyl, *N*,*N*-dimethylamino and ethyl groups. Their properties as hole transport materials in perovskite solar cells are investigated. The impact of replacing the *para*-methoxy substituent on bulk properties, such as the photophysical properties, HOMO/LUMO energy level, hole extraction properties and morphologies of perovskite thin films are investigated. Their optoelectronic and charge-transport properties and performance in perovskite solar cells are compared with the current benchmarked and structurally-related hole transport material (HTM) Spiro-MeOTAD. Surprisingly, the methylsulfanyl substituted spirofluorene shows the highest power conversion efficiency of 15.92% among the investigated spirofluorenes, which is an over 38% increase in PCE compared with that of Spiro-MeOTAD under similar device fabrication conditions.

## Abstract 2

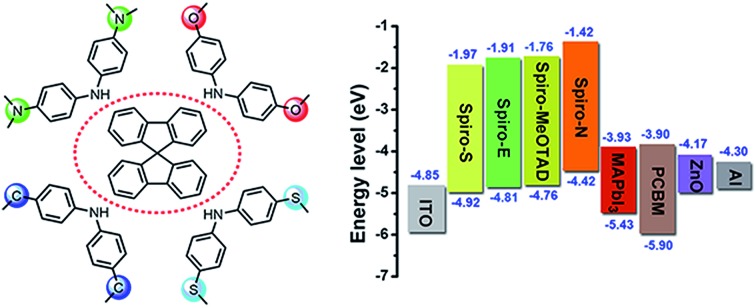
By introducing the heteroatom into the benchmark hole transport material Spiro-MeOTAD, the energy level of hole transport materials can be tuned.

## 

Charge transport materials hold the key to the fabrication of high performance devices. In 1987, a double layer OLED using thin films of 1,1-bis{4-[di(*p*-tolyl) amino]-phenyl}·cyclohexane (TAPC) as a hole transport material was reported and a significant decrease in its drive voltage was shown.^1^,^2^ Since the discovery by Grätzel in 1991,^3^ dye-sensitized solar cells (DSSCs) have attracted a great deal of interest due to their low-cost materials, solution processibility and low energy expenditure.^4^–^6^ However, the best-performing solid-state dye-sensitized solar cell (ssDSSC) still had a power conversion efficiency (PCE) below 7% (ref. 7) until organic–inorganic perovskite compounds were utilized as sensitizers. CH_3_NH_3_PbI_3_ (MAPbI_3_) and CH_3_NH_3_PbBr_3_ (MAPbBr_3_) were used, for the first time, as the light absorbers in liquid dye-sensitized solar cells by Miyasaka *et al.*^8^ Subsequent to this breakthrough, a lead halide perovskite-based ssDSSC coupled with Spiro-MeOTAD as a HTM achieved much higher PCEs than those of liquid ones, rapidly surpassing the 15% barrier.^9^–^14^ A PCE of over 20% has been reported^15^–^17^ and a record PCE of 22.1% has been confirmed.^18^ Until now, Spiro-MeOTAD has been the main HTM studied in perovskite solar cells (PSCs). Actually, the best performance of almost all the reported highly efficient devices has been achieved with Spiro-MeOTAD as a HTM in both perovskite-based and dye-sensitized solar cells.^13^,^19^–^24^ Several attempts to improve organic HTMs for DSSCs have been reported. For example, P3HT, PCBTDPP, PCPDTBT and PTAA were employed for the fabrication of perovskite solar cells.^25^ However, using polymers as the HTMs creates potential problems related to batch-to-batch variation and difficulties in purification, for example. Several small molecule HTMs^26^,^27^ and certain highly-efficient small molecule donors^28^,^29^ used in bulk heterojunction solar cells have been applied as HTMs to achieve high PCEs. For example, as reported recently by Jeon *et al.*, changing the *p*-OMe substituents to the *ortho*-position of the conventional Spiro-MeOTAD leads to improved efficiency of up to 16.7% in the *meso*-structured perovskite solar cells.^14^

In optoelectronic materials, sulphur- and nitrogen-substituents are often used as means to tune the frontier orbital energy levels.^30^–^36^ Herein, we present three new hole transport materials derived from Spiro-MeOTAD by replacing the *p*-methoxy group with methylsulfanyl (Spiro-S), *N*,*N*-dimethylamino (Spiro-N) and ethyl (Spiro-E) groups. The molecular structures of the reported new HTMs and Spiro-MeOTAD are shown in Fig. 1 and their detailed synthetic procedures are provided in the ESI, Scheme 1.† The synthesis of intermediate **7** was quite difficult as we faced a low yield issue despite various palladium catalysts, ligands, and solvents being evaluated (see Fig. S1†). Finally, we found the combination of (DPPF)PdCl_2_–CH_2_Cl_2_, DPPF, and toluene to afford a high yield of over 60%.

**Fig. 1:**
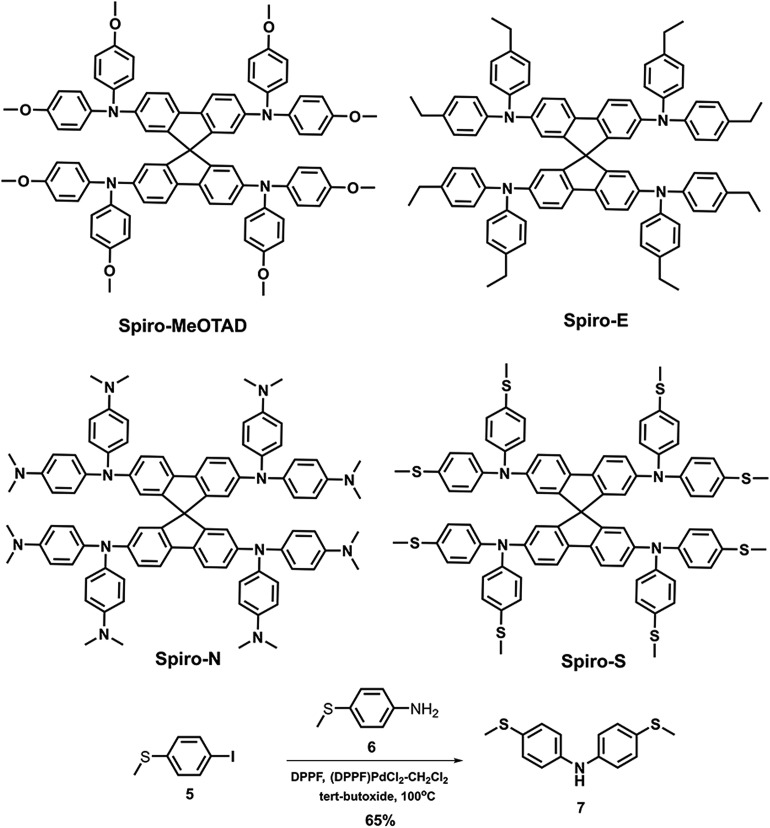
Chemical structures of the HTMs.

To gain insight into the influence of the chemical structure on the electrochemical properties of these materials, cyclic voltammetry was employed to measure the electronic energy levels of the HTMs. The HOMO energy levels were calculated from the onset oxidation potential by assuming the energy level of ferrocene/ferrocenium (Fc/Fc^+^) to be –4.8 eV below the vacuum level.^37^Fig. 2a displays the cyclic voltammograms of ferrocene and the three HTMs in Bu_4_NPF_6_ (0.1 M in CH_2_Cl_2_) solution at a scan rate of 100 mV s^–1^. The onset oxidation potentials of Spiro-MeOTAD, Spiro-S, Spiro-N and Spiro-E are 0.165, 0.331, 0.169, and 0.220 V (*vs.* Ag/Ag^+^), respectively. The potential of Fc/Fc^+^ was 0.21 V (*vs.* Ag/Ag^+^) as measured in our study and therefore the HOMO energy levels of these HTMs were calculated accordingly as –4.76, –4.92, –4.42 and –4.81 eV for Spiro-MeOTAD, Spiro-S, Spiro-N and Spiro-E, respectively. The corresponding UV-vis absorption spectra of the HTMs in CHCl_3_ (10^–5^ M) and in solid film are displayed in Fig. 2b. The optical band gaps estimated from the onset of the absorption (in solution) are 3.0 eV for Spiro-MeOTAD, 2.90 eV for Spiro-E, 3.0 eV for Spiro-S and 2.84 eV for Spiro-N. The LUMO energy levels were calculated to be –1.76, –1.97, –1.42 and 1.81 eV for Spiro-MeOTAD, Spiro-S, Spiro-N and Spiro-E, respectively. Spiro-N is found to have a higher HOMO energy level due to the strong electron-donating ability of the two *N*,*N*-dimethylamino groups. Compared to Spiro-MeOTAD, the HOMO level of Spiro-S with methylsulfanyl groups decreased by 0.16 eV and Spiro-E with ethyl groups by 0.05 eV, indicating the significant influence of these *para*-substituents on the electronic properties. Thus, the energy levels of Spiro-MeOTAD-based HTMs could be easily tuned, as shown in Fig. 3b.

**Fig. 2:**
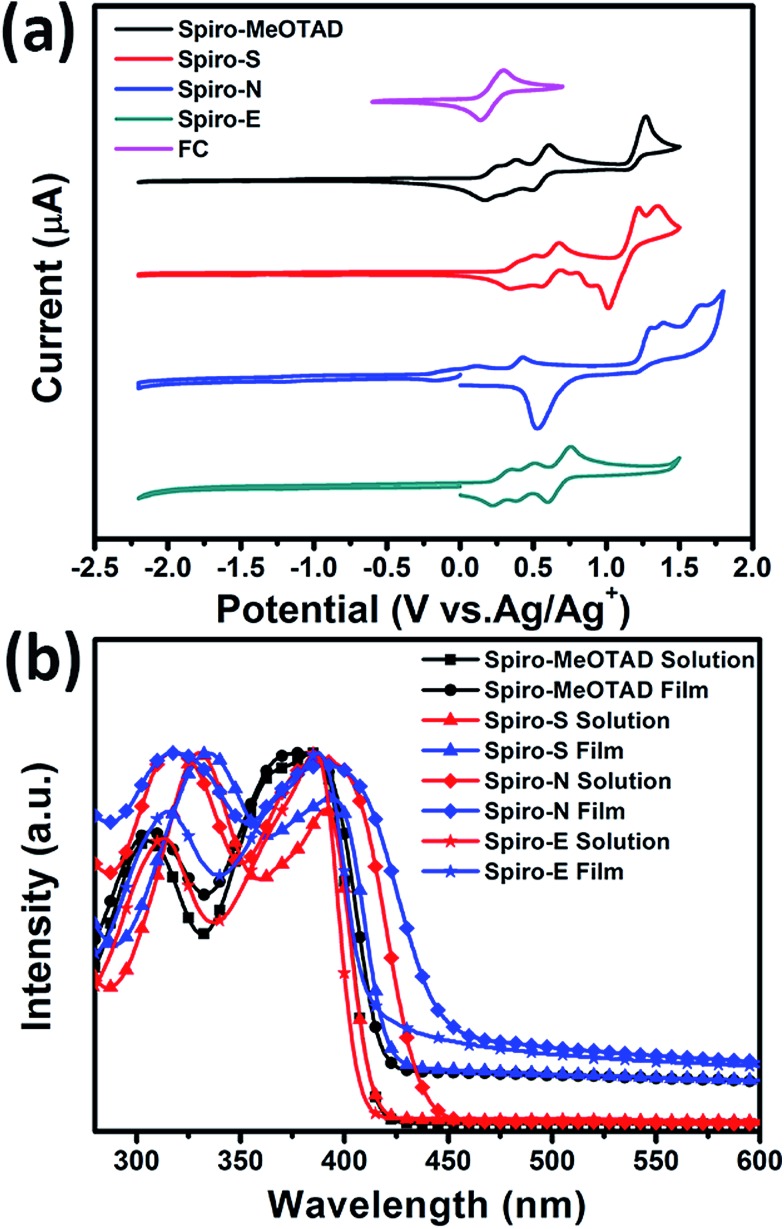
(a) Cyclic voltammograms of Spiro-MeOTAD, Spiro-S, Spiro-N, Spiro-E and ferrocene. (b) UV-vis spectra of the HTMs in solution and thin film.

**Fig. 3:**
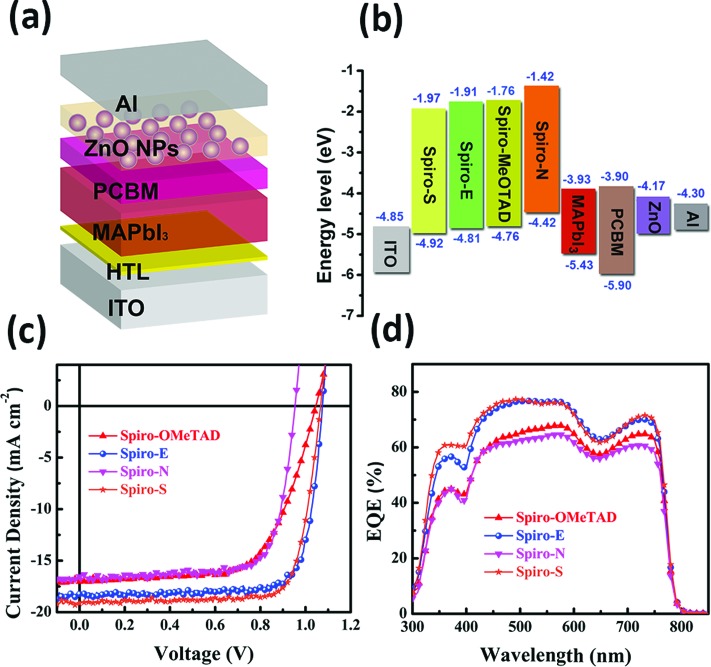
(a) The structure of the perovskite solar cell devices; (b) energy level diagram of the tested HTMs; (c) photocurrent density–voltage (*J*–*V*) curves and (d) EQE spectra of the corresponding solar cells.

Thermogravimetric analysis (TGA) (see ESI Fig. S2a†) reveals the decomposition temperature of Spiro-S to be at 372 °C, similar to that of Spiro-MeOTAD (400 °C), while Spiro-N and Spiro-E show higher decomposition temperatures of 426 °C and 430 °C, respectively, as compared to Spiro-MeOTAD. Hence, both Spiro-N and Spiro-E show higher thermal stability. Differential scanning calorimetry (DSC) analyses (see ESI Fig. S2b†) were performed on the four materials. For Spiro-MeOTAD and Spiro-E, both glass transition (at 158 °C, –33.54 J g^–1^ and 255 °C, –7.28 J g^–1^) and melting (at 245 °C, –50.34 J g^–1^ and 267 °C, –33.4 J g^–1^) are observed. In addition, crystallization processes are detected at 180 °C (35.55 J g^–1^) for Spiro-MeOTAD and at 190 °C (4.21 J g^–1^) and 203 °C (8.78 J g^–1^) for Spiro-E. No obvious peak of glass transition for Spiro-S and Spiro-N is detected. Instead, crystallization processes are detected at 174 °C (9.14 J g^–1^) and 215 °C (20.27 J g^–1^) for Spiro-S and Spiro-N, respectively. The melting temperatures of the corresponding crystals are 252 (–18.59 J g^–1^) and 316 °C (–37.13 J g^–1^) for Spiro-S and Spiro-N, respectively. This indicates that, similar to Spiro-MeOTAD, Spiro-S, Spiro-N and Spiro-E can also have both amorphous and crystalline forms.

The charge transport properties of the four HTMs have been investigated using the space-charge-limited-current (SCLC) technique (Fig. S3†);^38^ the mobility data is listed in Table 1. Spiro-MeOTAD and Spiro-E show quite similar values of hole mobility. However, the hole mobility of Spiro-S is slightly higher than that of Spiro-MeOTAD, while Spiro-N shows the lowest mobility.

**Table 1:** The hole mobility of different HTMs^*a*^

HTM	*μ*_h_ (×10^–5^ cm^2^ V^–1^ s^–1^)
Spiro-MeOTAD	1.55 ± 0.28
Spiro-E	1.26 ± 0.21
Spiro-N	0.25 ± 0.04
Spiro-S	1.90 ± 0.38

^*a*^The values were calculated by averaging data collected from eight devices.

In order to evaluate the three new spiro-bifluorene-based materials as promising hole transport materials for perovskite solar cells, we fabricated perovskite solar cells with the device structure of ITO/HTM/CH_3_NH_3_PbI_3_/PCBM/ZnO nanoparticles/Al, as shown in Fig. 3a.^17^ A layer of ZnO nanoparticles was applied as the ohmic contact to assist the electron extraction from PCBM to Al. For comparison, devices with Spiro-MeOTAD as the HTM were also fabricated and tested (the detailed device fabrication processes are provided in the ESI†). The photocurrent density–voltage (*J*–*V*) curves of the devices with different HTMs are shown in Fig. 3c and the relevant photovoltaic data of the devices are summarized in Table 2.

**Table 2:** Summary of photovoltaic parameters of the solar cells using different HTMs

HTL	*V*_oc_	*J*_sc_	FF	PCE	Calculated *J*_sc_ (mA cm^–2^)
Spiro-MeOTAD	1.04 (1.00 ± 0.04)	17.04 (17.42 ± 0.55)	0.65 (0.60 ± 0.03)	11.55 (10.37 ± 0.54)	15.55
Spiro-E	1.07 (1.07 ± 0.01)	18.24 (18.43 ± 0.32)	0.80 (0.74 ± 0.04)	15.75 (14.63 ± 0.70)	17.57
Spiro-N	0.96 (0.92 ± 0.02)	16.55 (16.09 ± 0.48)	0.75 (0.74 ± 0.02)	11.92 (10.95 ± 0.75)	14.88
Spiro-S	1.06 (1.05 ± 0.03)	19.15 (18.64 ± 0.43)	0.78 (0.77 ± 0.01)	15.92 (15.08 ± 0.50)	17.75

While the best PCE of the Spiro-MeOTAD-based device is 11.55%, the PCEs of the corresponding perovskite solar cells made of Spiro-E, Spiro-N, and Spiro-S are 15.75%, 11.92%, and 15.92%, respectively. The devices with Spiro-E and Spiro-S show higher open circuit voltage (*V*_oc_ = 1.07 V for Spiro-E and 1.06 V for Spiro-S) compared to that of the Spiro-MeOTAD (1.04 V). The slightly higher values from Spiro-E and Spiro-S are due to their lower HOMO energy level as compared to Spiro-MeOTAD (Fig. 3b),^39^ while the lower *V*_oc_ of the Spiro-N-based device is attributed to its higher HOMO energy level. The short circuit current densities (*J*_sc_) of the best devices fabricated with Spiro-E, Spiro-S, Spiro-N and Spiro-MeOTAD are 18.24, 19.15, 16.55 and 17.07 mA cm^–2^, respectively. The *J*_sc_ values are well matched with the integrated *J*_sc_ values obtained from the EQE spectra as shown in Fig. 3d and also show similar trends.

In order to better understand the effects and relationship of molecular structure and device performance, we conducted an in-depth morphological study. The top-view scanning electron microscopy (SEM) images of MAPbI_3_ films on various HTMs shown in Fig. 4a–d reveal a clear correlation between the substrate surface and the film grain morphology. The grain size for MAPbI_3_ on films made of Spiro-MeOTAD, Spiro-E and Spiro-S is obviously larger than that on Spiro-N. Furthermore, the contact angle tests (see ESI†) indicated that Spiro-N exhibits a smaller contact angle (70.0°) than that of Spiro-MeOTAD (78.7°). In contrast, Spiro-E and Spiro-S exhibit much larger contact angles of 82.5° and 91.5°, respectively. The more hydrophobic surface contributes to the larger grain size and better quality perovskite polycrystalline films.^40^

**Fig. 4:**
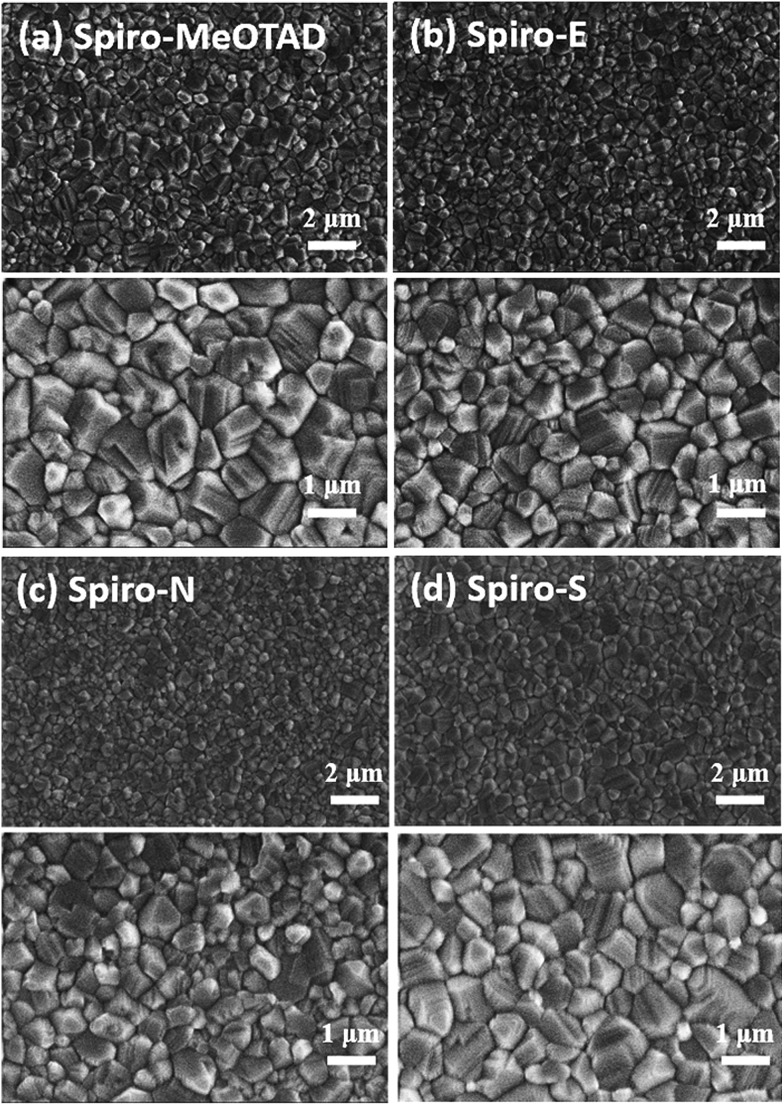
Top-view SEM of (a) Spiro-MeOTAD, (b) Spiro-E, (c) Spiro-N, and (d) Spiro-S.

Fig. 5a shows the photoluminescence (PL) spectra of perovskite on different HTM substrates. An enhanced PL quenching effect was observed for the substrates made of Spiro-E, Spiro-N and Spiro-S compared to those of Spiro-MeOTAD. This indicates that carriers created in the excited perovskite layers are extracted more efficiently by Spiro-E and Spiro-S. However, the strongest extraction ability observed in the Spiro-N films is somewhat contradictory with the SCLC hole mobility observed in the HTMs. Time-resolved PL (TRPL) measurements were conducted to verify the improved hole transfer phenomena, as shown in Fig. 5b. The results are summarized in Table S1 (see ESI†). The perovskite films made of Spiro-MeOTAD exhibit an average decay time (average *τ*) of 131.66 ns. When using Spiro-E, Spiro-N and Spiro-S as the substrate, the average *τ* is shortened to 67.04, 8.48 and 53.45 ns, respectively. From these observations, the unusual extraction ability of Spiro-N may be attributed to the tertiary amino groups as efficient hole trappers, which suppress the hole carrier transportation and result in localized recombination centers at the Spiro-N surface.^33^ Nevertheless, Spiro-E and Spiro-S also demonstrate better hole extraction capability and charge dissociation compared to those of Spiro-MeOTAD.

**Fig. 5:**
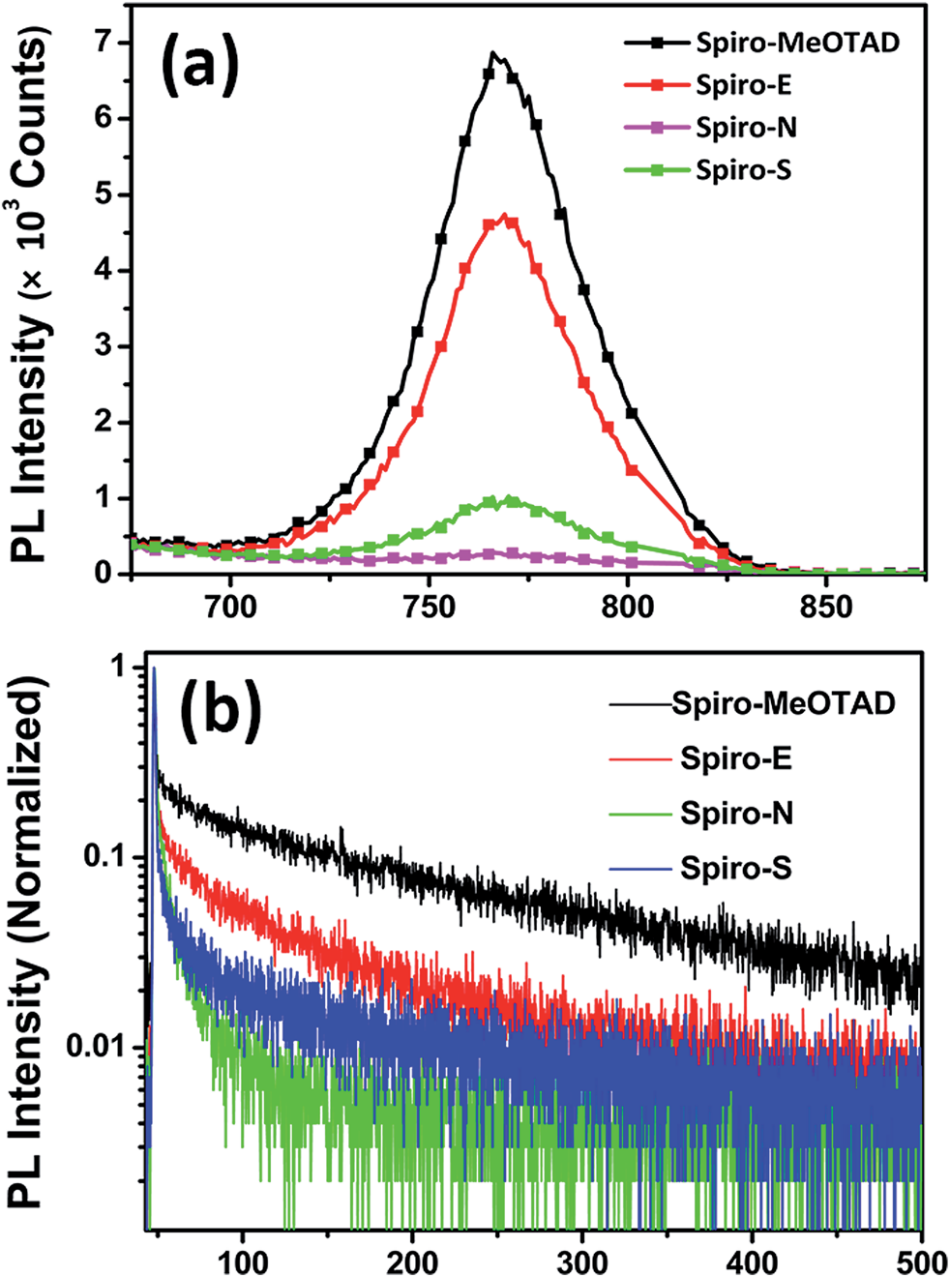
(a) Steady state PL spectra of the perovskite films on different HTM substrates; (b) time-resolved PL measurements taken at the peak emission wavelength (765 nm) of the perovskite films on different HTM substrates.

In conclusion, we have successfully synthesized and characterized three new spiro-bifluorenes as hole transport materials. The influence of heteroatom substitution on the optoelectronic properties, power conversion efficiency and charge-transport behaviour in perovskite solar cells is studied. Compared with Spiro-MeOTAD, Spiro-E and Spiro-S show closer HOMO energy levels which are well matched with that of CH_3_NH_3_PbI_3_. Moreover, Spiro-E and Spiro-S also exhibited greater hydrophobicity, which contributes to the larger grain size and better quality perovskite polycrystalline films. A perovskite solar cell with a high PCE of 15.92% is obtained for the Spiro-S-based devices. As a good example, our results open up the potential incorporation of heteroatoms into new hole transport materials to develop better perovskite solar cells in the future.

## Supplementary Material

Supplementary information

## References

[cit1] Tang C. W., VanSlyke S. A. (1987). Appl. Phys. Lett..

[cit2] Tang C. W., VanSlyke S. A., Chen C. H. (1989). J. Appl. Phys..

[cit3] O'Regan B., Grätzel M. (1991). Nature.

[cit4] Hashmi G., Miettunen K., Peltola T., Halme J., Asghar I., Aitola K., Toivola M., Lund P. (2011). Renewable Sustainable Energy Rev..

[cit5] Crossland E. J. W., Noel N., Sivaram V., Leijtens T., Alexander-Webber J. A., Snaith H. J. (2013). Nature.

[cit6] Yamaguchi T., Tobe N., Matsumoto D., Nagai T., Arakawa H. (2010). Sol. Energy Mater. Sol. Cells.

[cit7] Burschka J., Dualeh A., Kessler F., Baranoff E., Cevey-Ha N.-L., Yi C., Nazeeruddin M. K., Grätzel M. (2011). J. Am. Chem. Soc..

[cit8] Kojima A., Teshima K., Shirai Y., Miyasaka T. (2009). J. Am. Chem. Soc..

[cit9] Burschka J., Pellet N., Moon S.-J., Humphry-Baker R., Gao P., Nazeeruddin M. K., Gratzel M. (2013). Nature.

[cit10] Liu D., Kelly T. L. (2014). Nat. Photonics.

[cit11] Wang J. T.-W., Ball J. M., Barea E. M., Abate A., Alexander-Webber J. A., Huang J., Saliba M., Mora-Sero I., Bisquert J., Snaith H. J., Nicholas R. J. (2014). Nano Lett..

[cit12] Wojciechowski K., Saliba M., Leijtens T., Abate A., Snaith H. J. (2014). Energy Environ. Sci..

[cit13] Liu M., Johnston M. B., Snaith H. J. (2013). Nature.

[cit14] Jeon N. J., Lee H. G., Kim Y. C., Seo J., Noh J. H., Lee J., Seok S. I. (2014). J. Am. Chem. Soc..

[cit15] Jesper Jacobsson T., Correa-Baena J.-P., Pazoki M., Saliba M., Schenk K., Grätzel M., Hagfeldt A. (2016). Energy Environ. Sci..

[cit16] Yang W. S., Noh J. H., Jeon N. J., Kim Y. C., Ryu S., Seo J., Seok S. I. (2015). Science.

[cit17] Bi D., Tress W., Dar M. I., Gao P., Luo J., Renevier C., Schenk K., Abate A., Giordano F., Correa Baena J.-P., Decoppet J.-D., Zakeeruddin S. M., Nazeeruddin M. K., Grätzel M., Hagfeldt A. (2016). Sci. Adv..

[cit18] Research Cell Efficiency Records, NREL, http://www.nrel.gov/ncpv/, accessed: April 2016.

[cit19] Yang L., Cappel U. B., Unger E. L., Karlsson M., Karlsson K. M., Gabrielsson E., Sun L., Boschloo G., Hagfeldt A., Johansson E. M. J. (2012). Phys. Chem. Chem. Phys..

[cit20] Jiang X., Karlsson K. M., Gabrielsson E., Johansson E. M. J., Quintana M., Karlsson M., Sun L., Boschloo G., Hagfeldt A. (2011). Adv. Funct. Mater..

[cit21] Cai N., Moon S.-J., Cevey-Ha L., Moehl T., Humphry-Baker R., Wang P., Zakeeruddin S. M., Grätzel M. (2011). Nano Lett..

[cit22] Snaith H. J., Grätzel M. (2007). Adv. Mater..

[cit23] Lee M. M., Teuscher J., Miyasaka T., Murakami T. N., Snaith H. J. (2012). Science.

[cit24] Noh J. H., Jeon N. J., Choi Y. C., Nazeeruddin M. K., Gratzel M., Seok S. I. (2013). J. Mater. Chem. A.

[cit25] Heo J. H., Im S. H., Noh J. H., Mandal T. N., Lim C.-S., Chang J. A., Lee Y. H., Kim H.-j., Sarkar A., NazeeruddinMd K., Gratzel M., Seok S. I. (2013). Nat. Photonics.

[cit26] Li H., Fu K., Hagfeldt A., Grätzel M., Mhaisalkar S. G., Grimsdale A. C. (2014). Angew. Chem., Int. Ed..

[cit27] Xu B., Sheibani E., Liu P., Zhang J., Tian H., Vlachopoulos N., Boschloo G., Kloo L., Hagfeldt A., Sun L. (2014). Adv. Mater..

[cit28] Zheng L., Chung Y.-H., Ma Y., Zhang L., Xiao L., Chen Z., Wang S., Qu B., Gong Q. (2014). Chem. Commun..

[cit29] Liu Y., Chen Q., Duan H.-S., Zhou H., Yang Y., Chen H., Luo S., Song T.-B., Dou L., Hong Z., Yang Y. (2015). J. Mater. Chem. A.

[cit30] Peng Q., Liu X., Su D., Fu G., Xu J., Dai L. (2011). Adv. Mater..

[cit31] Kan B., Zhang Q., Li M., Wan X., Ni W., Long G., Wang Y., Yang X., Feng H., Chen Y. (2014). J. Am. Chem. Soc..

[cit32] Huo L., Zhou Y., Li Y. (2009). Macromol. Rapid Commun..

[cit33] Duan C., Cai W., Hsu B. B. Y., Zhong C., Zhang K., Liu C., Hu Z., Huang F., Bazan G. C., Heeger A. J., Cao Y. (2013). Energy Environ. Sci..

[cit34] Cui C., Wong W.-Y., Li Y. (2014). Energy Environ. Sci..

[cit35] Lee R.-H., Cheng T.-F., Chang J.-W., Ho J.-H. (2011). Colloid Polym. Sci..

[cit36] Zhou G., Pschirer N., Schöneboom J. C., Eickemeyer F., Baumgarten M., Müllen K. (2008). Chem. Mater..

[cit37] Pommerehne J., Vestweber H., Guss W., Mahrt R. F., Bässler H., Porsch M., Daub J. (1995). Adv. Mater..

[cit38] Poplavskyy D., Nelson J. (2003). J. Appl. Phys..

[cit39] Rakstys K., Abate A., Dar M. I., Gao P., Jankauskas V., Jacopin G., Kamarauskas E., Kazim S., Ahmad S., Grätzel M., Nazeeruddin M. K. (2015). J. Am. Chem. Soc..

[cit40] Bi C., Wang Q., Shao Y., Yuan Y., Xiao Z., Huang J. (2015). Nat. Commun..

