# Supplementary material for: Effects of heteroatom substitution in spiro-bifluorene hole transport materials
Source: Chem Sci. 2016 May 3;7(8):5007–12. doi: 10.1039/c6sc00973e (PMC6018644; doi:10.1039/c6sc00973e)
Supplement: Supplementary file 1 [file SC-007-C6SC00973E-s001.pdf]

Electronic Supplementary Material (ESI) for Chemical Science.
This journal is © The Royal Society of Chemistry 2016

Supplementary Information

Effects of Heteroatom Substitution in Spiro-bifluorene Hole Transport Materials

Zhao Hu,[a] Weifei Fu,[b] Lijia Yan,[c] Jingsheng Miao,[a] Hongtao Yu,[b] Yaowu He,[a] Osamu Goto,[a] Hong Meng,[a,c,*] Hongzheng Chen,[b*] Wei Huang.[c*]

1. Synthesis of HTMs

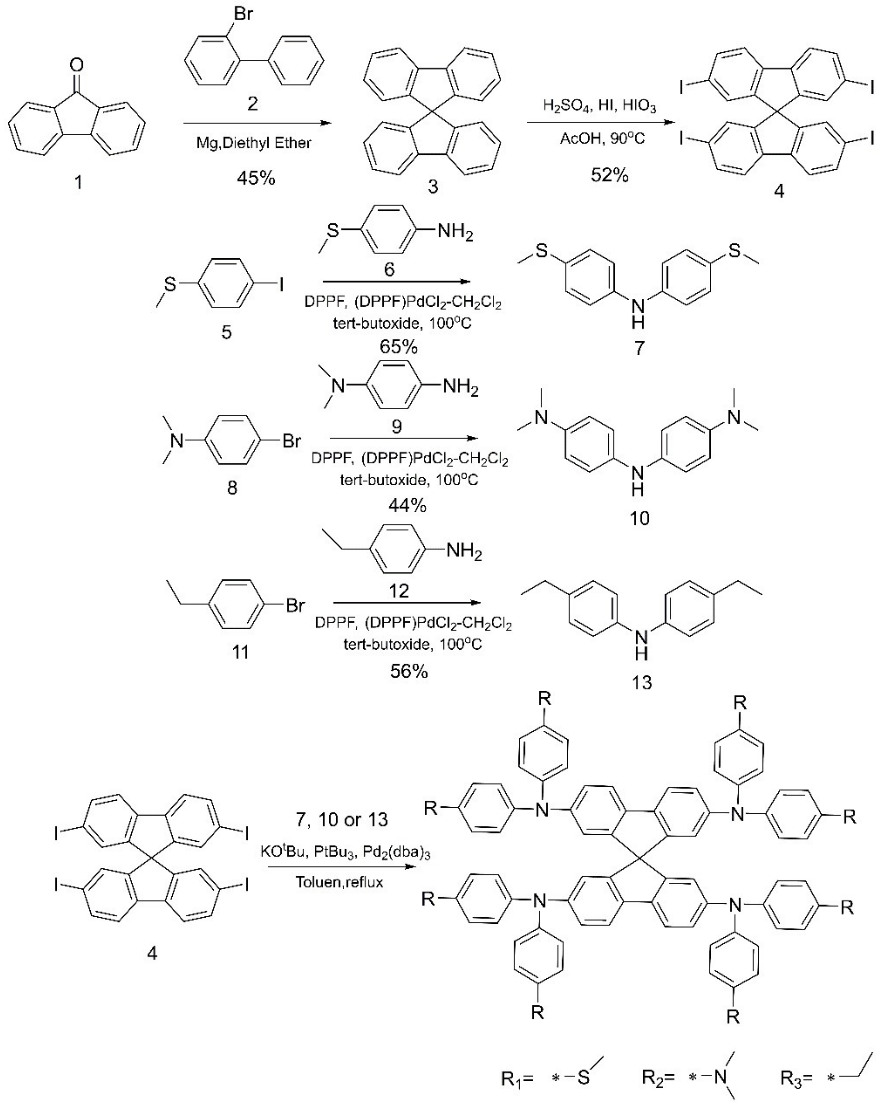

Scheme 1. Synthetic route of Spiro-S, Spiro-N and Spiro-E

The representative synthesis process of Spiro-S and Spiro-N is shown in in Scheme 1. All starting materials were obtained from commercial suppliers without further purification. And all metal catalysts were purchased from TCI. 9, 9'-spriobifluorene (3), 2, 2', 7, 7'-tetraiodo-9, 9'-spirobifluorene (4) and N^1-(4-(dimethylamino) phyenyl)-N^4, N^4-dimethylbenzene-1, 4-diamine (10) were synthesized according to the literature procedures.[1,2,3]

General Method for the preparation of the Diarylamines

A nitrogen flushed round bottom flask was charged with Aryl bromide (1 mmol), arylamine (1.1 mmol) DPPF (0.12mmol), (DPPF) $PdCl_2$-CH_2Cl_2 (0.004mmol) and potassium tert-butoxide (1.4 mmol). Toluene was then added and mixture was bubbled with nitrogen for 15mim. The mixture was refluxed for 24h before quenching by water. Then, the solution was extracted for three times and the organic layer was dried with Na_2SO_4 for two hours. After removal of solvent, the crude product was purified by column chromatography on silica gel.

Synthesis of bis (4-methylthiophenyl) amine (7)

Yield 65% yellow solid. ^{1}H NMR (CDCl3, 300MHz): δ 7.25 (M, 4H), 6.99 (d, *J*=8.4Hz 4H), 5.65 (s, 1H), 2.47 (s, 6H); ^{13}C NMR (CDCl3, 300MHz) δ 141.13, 129.77, 129.18, 118.45, 17.76.

Synthesis of N^1-(4-(dimethylamino) phyenyl)-N^4, N^4-dimethylbenzene-1, 4-diamine (10)

Yield 44% brown solid. ^{1}H NMR (acetone-*d6* 400MHz) δ 6.69 (d, *J*=8.3Hz, 4H), 6.70 (dt, *J*=8.3, 2.3Hz, 4H), 6.41 (brs, 1H), 2.82 (s, 12H).

Synthesis of 4-ethyl-N-(4-ethylphenyl) (13)

Yield 55.6% green solid. ^{1}H NMR (CDCl3, 400MHz): δ 7.15 (d, *J*=12 Hz, 8H), 7.03 (d, *J*=8.4 Hz, 4H), 5.58 (s, 1Hz), 2.65(m, 4H), 1.28(m, 6H); ^{13}C NMR (CDCl3, 300MHz) δ 141.43, 136.73, 128.75, 118.01, 28.29, 16.01.

General Method for the preparation of Spiro-bifluorene Hole Transport Materials

A nitrogen flushed round bottom flask was charged with diarylamine (5mmol), 2, 2', 7, 7'-tetraiodo-9, 9'-spirobifluoren (1mmol) $Pd_2(dba)_3$ (0.05mmol), potassium tert-butoxide (5mmol) and $PtBu_3$ (0.1mmol). Toluene was then added and the mixture was bubbled with nitrogen for 15mim. The mixture was refluxed for 24h before quenching by water. Then, the solution was extracted with ethyl acetate for three times and organic layer was dried with Na_2SO_4 for two hours. After removal of solvent, the crude product was purified by column chromatography on silica gel.

Synthesis of Spiro-S

Yield 52% yellow crystals. ^{1}H NMR (DMSO-*d6*, 300 MHz): δ 7.63 (d, *J* = 8.4 Hz, 4H), 7.14 (d, *J* = 8.7 Hz, 16H), 6.88 (dd, *J* = 8.3, 2.1, 4H), 6.82 (d, *J* = 8.7, 16H), 6.33 (d, *J* = 2.1, 4H), 2.42 (s, 24H); ^{13}C NMR (DMSO-*d6*, 300MHz) δ 149.71, 145.53, 144.68, 136.00, 132.08, 128.04, 124.32, 15.84. MS (MALDI-TOF) m/z: calculated for $C_{81}H_{68}N_4S_8$, $[M]^+$, 1352.3210; found, 1352.3279

Synthesis of Spiro-N

Yield 45% brown solid. ^{1}H NMR (DMSO-*d6*, 300 MHz): δ 7.33 (s, 4H), 6.69 (M, 40H), 3.35 (s, 48H); 13C NMR (DMSO-d6, 300MHz) δ MS (MALDI-TOF) m/z: calculated for $C_{89}H_{92}N_{12}$, $[M]^+$, 1328.7568; found, 1328.7628

Synthesis of Spiro-E

Yield 55% green crystals. ^{1}H NMR (acetone-*d6*, 300 MHz): δ 7.57 (d, 6Hz, 4H), 7.12 (d, 6.6Hz, 15H), 6.91 (d, 6.3Hz, 17H), 6.88 (d, 1.5Hz, 2H), 6.59 (d, 1.5Hz, 4H), 2.8(s, 4H), 2.61 (m, 16H), 1.21(t, 24H); 13C NMR (CHCl3, 300MHz) δ 149.92, 146.76, 145.62, 137.96, 128.31, 124.44, 122.24, 123.24, 120.07, 119.72, 28.14, 15.53. MS (MALDI-TOF) m/z: calculated for $C_{89}H_{84}N_4$, $[M]^+$, 1208.6696; found 1208.6752

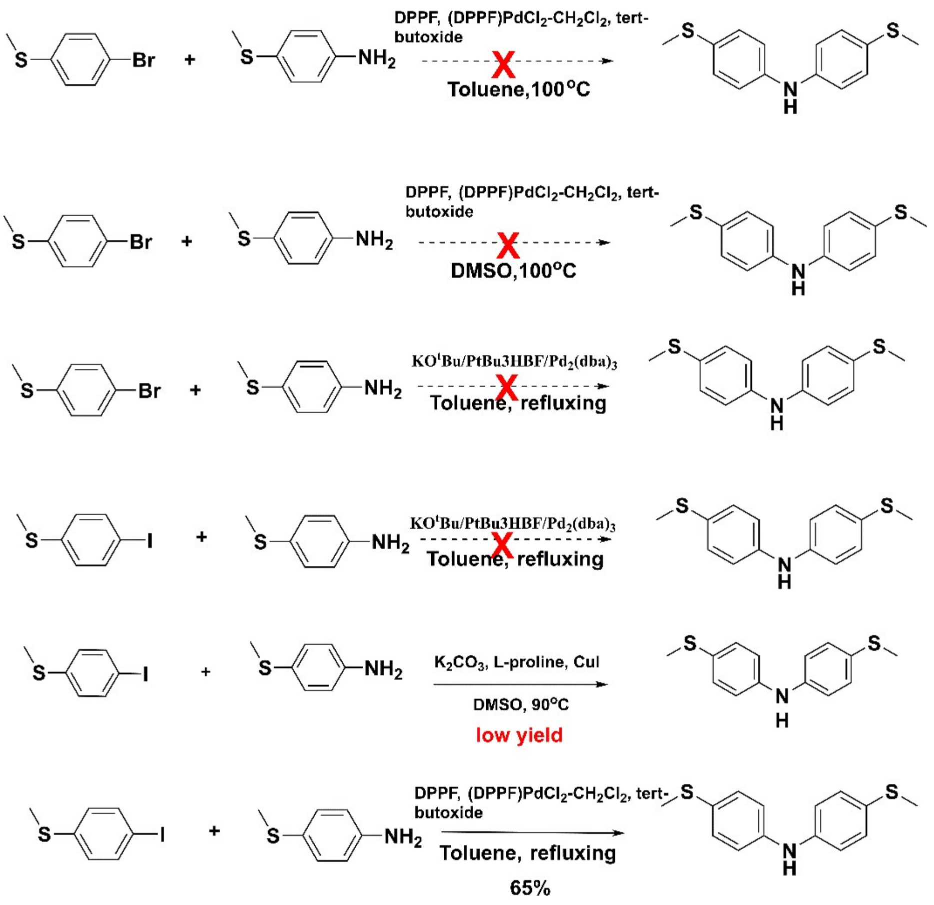

Figure. S1 Different synthesis conditions of the intermediate 7

2. Measurements

Nuclear magnetic resonance (NMR) was taken on Bruker AVANCE III 300 MHz and 400MHz Spectrometer. All chemical shifts were reported relative to tetramethylsilane (TMS) at 0.0ppm, unless otherwise stated.

High resolution mass spectra were obtained from a MALDI-TOF Mass Spectrometer.

Thermo-gravimetric analysis (TGA) and Differential scanning calorimetry (DSC) were carried out with a Mettler-Toledo TGA/DSC thermal analyzer under purified nitrogen gas flow with a 10°C min^{-1} heating rate.

The absorption spectrum was recorded with UV-visible spectrophotometer (Shimadzu 2450). Cyclic voltammetry was determined by electrochemical workstation (Chenhua CHI600E).

The morphology was measured using Atomic Force Microscope (Bruker Multimode 8). All images were obtained using typical parameters for tapping mode imaging.

The SEM data were scanned by S-4800 (Hitachi) field-emission scanning electron microscope.

The PL spectra (both of steady state and transient spectra) are measured using an FluoroMax-4 HORIBA Jobin Yvon spectrofluorometer.

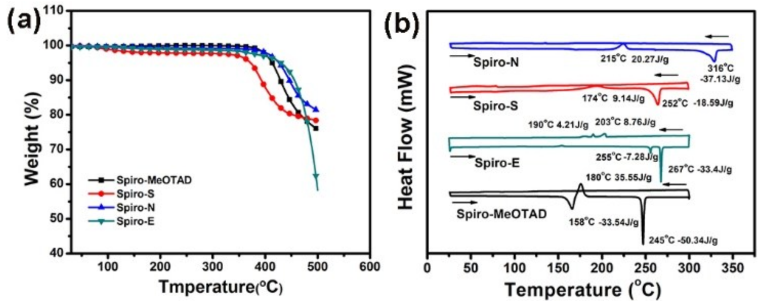

Figure S2. (a) Thermogravimetry plots of the HTMs (b) DSC curves of the HTMs with a heating rate 10°C min^{-1} under inert atmosphere

The charge carrier mobility of the HTMs films were measured using the space-charge-limited current (SCLC) method. The devices were fabricated in a structure of ITO/PEDOT:PSS/HTMs(50nm)/MoO_3/Al. The device measurements were extracted by modeling the dark current under forward bias using the SCLC expression describled by the Mott-Gurney law:

$$J = \frac{9}{8}\varepsilon_{\gamma}\varepsilon_0\mu\frac{V^2}{L^3}$$

Here, $\varepsilon_{\gamma} \approx 3$ is the average dielectric constant of the film, ε_0 is the permittivity of the free space, μ is the carrier mobility, L ≈ 50nm is the thickness of the film and V is the applied voltage.

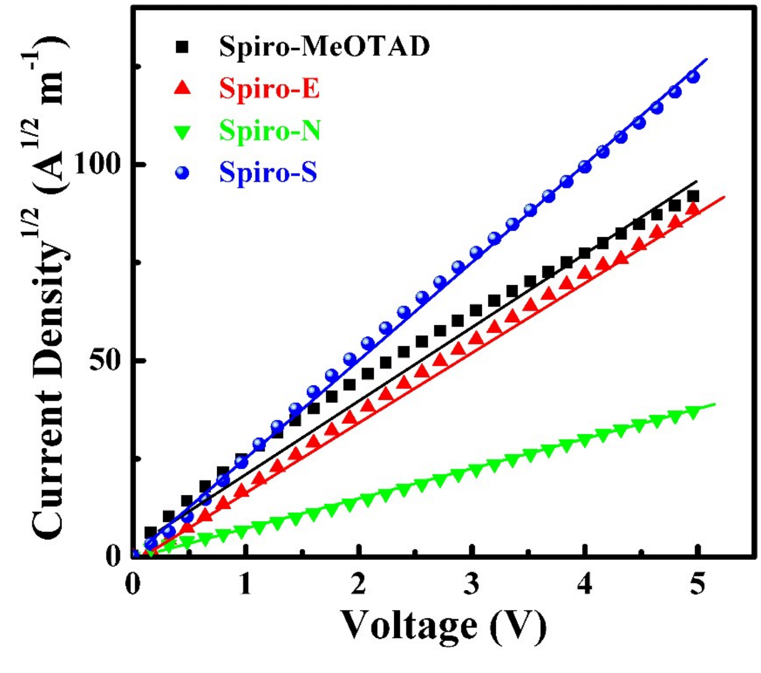

Figure S3. $J^{1/2}$-V curves of the hole-only devices with a architecture of ITO/PEDOT:PSS/HTMs/MoO_3/Al

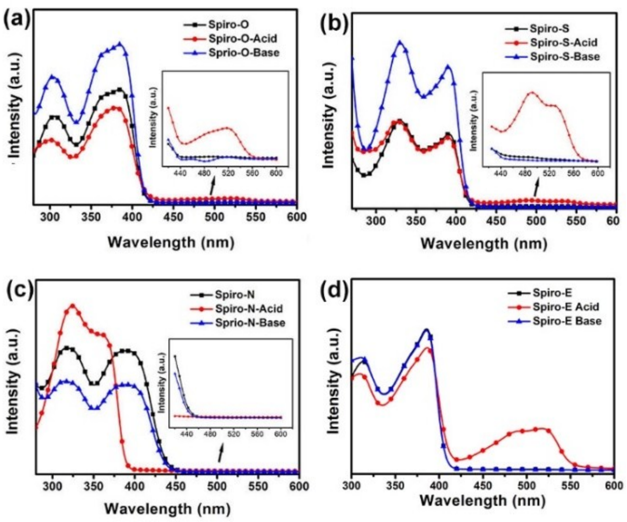

Figure S4. (a) UV-vis absorption spectra of the Spiro-MeOTAD added acid and base. (b) UV-vis absorption spectra of the Spiro-S added acid and base. (c) UV-vis absorption spectra of the Spiro-N added acid and base. (d) UV-vis absorption spectra of the Spiro-E added acid and base.

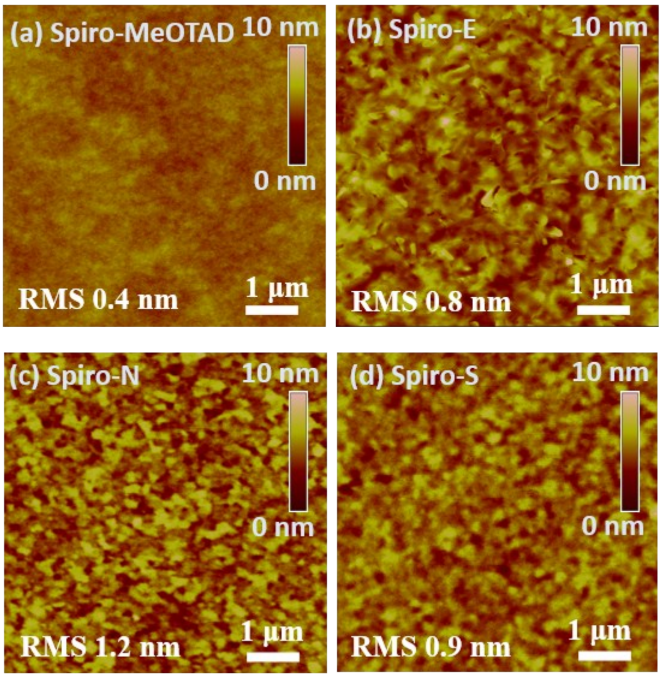

Figure S5. AFM images of HTMs films

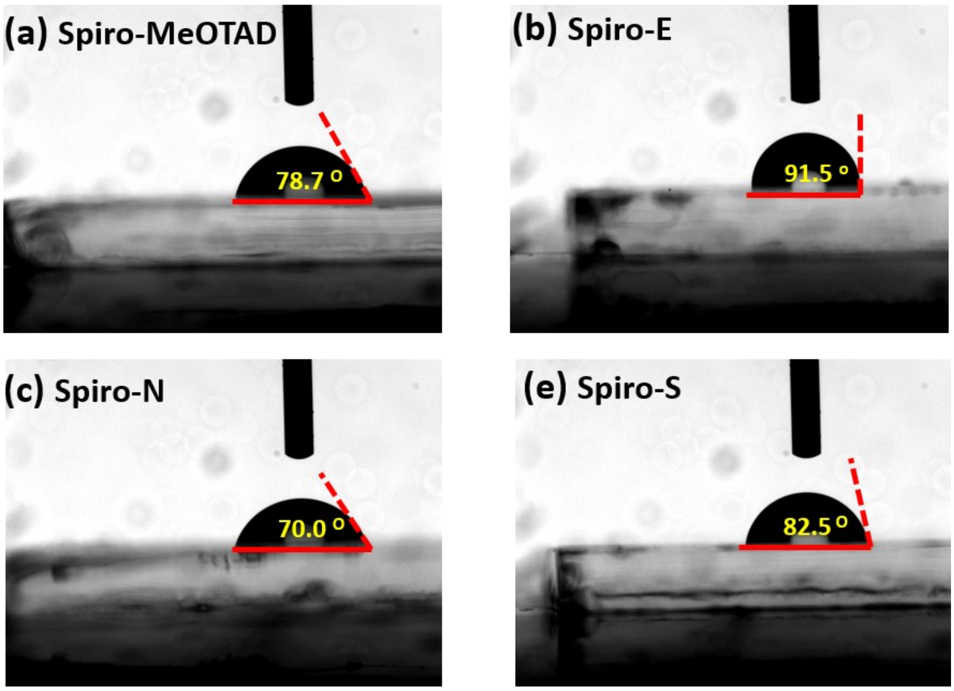

Figure S6. Water contact angles on (a) Spiro-MeOTAD, (b) Spiro-E, (c) Spiro-N, (d) Spiro-S

Table S1. Time-Resolved PL Measurements

sample	τ_1 (ns)	frac. 1	τ_2 (ns)	frac. 2	ave. (ns)
Spiro-MeOTAD	0.93	2.64%	135.2	97.36%	131.66
Spiro-E	0.79	11.68%	75.8	88.32%	67.04
Spiro-N	0.91	41.30%	13.8	58.70%	8.48
Spiro-S	0.87	29.05%	75.0	70.95%	53.45

3. The perovskite solar cells devices fabrication and testing

All reagents and solvents, unless otherwise specified, were purchased from Aladdin, Aldrich and J&K Scientific Ltd. and were used without further purification. $PC_{71}BM$ was purchased from American Dyes Source, Inc. CH_3NH_3I (MAI) was purchased from Shanghai Materwin New Materials Co. Ltd.

Prior to fabrication, the substrates were cleaned by sonication using detergent, deionized water, acetone, and isopropanol sequentially for every 15 min followed by 15 min of ultraviolet ozone (UV-Ozone) treatment. The substrates were transferred to a glove box. Then a layer of HTL (5 mg mL^{-1} in toluene) was spin-coated onto the cleaned ITO and annealed at 110°C for 10 min.

PbI_2 was dissolved in DMF at a concentration of 1 M under stirring at 70°C with 1 mole DMSO. The solution was kept at 70°C during the whole procedure. The solution was then spin coated on the as-prepared HTL film at 6000 rpm for 60 s. The films were dropped with a solution of MAI in 2-propanol (IPA) (50 mg mL^{-1}) and spin-coated at 6000 rpm for 60 s. Afterwards, the as prepared films were heated at 90°C for 15 min. An electron transporting layer (ETL) was then deposited via spin coating a layer of [6,6]-phenyl-C_{61}-butyric acid methyl ester ($PC_{61}BM$, 20 mg mL^{-1}) in chlorobenzene at 2000 rpm for 45s. A hole-blocking layer was deposited via spin-coating

ZnO nanoparticles in ethanol at 4000 rpm for 30 s on the top of $PC_{61}BM$ layer. Subsequently, samples were loaded into a vacuum deposition chamber (background pressure ≈ 5×10^{-4} Pa) to deposit a 100 nm thick Al cathode with a shadow mask defining an active device area of 5.5 mm^2.

The *J-V* characteristics were measured with Keithley 2400 measurement source units with the devices maintained at room temperature in air. The photovoltaic response was measured under a calibrated solar simulator (Abet 300 W) at 100mWcm^{-2}, and the light intensity was calibrated with a standard photovoltaic reference cell. The EQE spectrum was measured using a Stanford Research System Model SR830 Lock-in Amplifier unit coupled with a monochromator and a 500W xenon lamp, and a calibrated Si photodiode with known spectral response was used as a reference.

References:

1. Pei, J.; Ni, J.; Zhou, X.-H.; Cao, X.-Y.; Lai, Y.-H., *J. Org. Chem.* 2002, 67, 4924.
2. Wu, R.; Schumm, J. S.; Pearson, D. L.; Tour, J. M., *J. Org. Chem.* 1996, 61, 6906.
3. Hanthorn, J. J.; Valgimigli, L.; Pratt, D. A., *J. Org. Chem.* 2012, 77, 6908.
